# Impaired Motor Recycling during Action Selection in Parkinson’s Disease

**DOI:** 10.1523/ENEURO.0492-19.2020

**Published:** 2020-04-27

**Authors:** Matthias Fritsche, Robrecht P. R. D. van der Wel, Robin Smit, Bastiaan R. Bloem, Ivan Toni, Rick C. Helmich

**Affiliations:** 1 Centre for Cognitive Neuroimaging, Donders Institute for Brain, Cognition and Behaviour, Radboud University, Nijmegen 6525 EN, The Netherlands; 2 Department of Psychology, Rutgers University, Camden, NJ 08102; 3 Donders Institute for Brain, Cognition and Behaviour; Department of Neurology; Center of Expertise for Parkinson & Movement Disorders, Radboud University Medical Center, Nijmegen 6525 GC, The Netherlands

**Keywords:** basal ganglia, motor efficiency, motor planning, Parkinson’s disease, priming

## Abstract

Behavioral studies have shown that the human motor system recycles motor parameters of previous actions, such as movement amplitude, when programming new actions. Shifting motor plans toward a new action forms a particularly severe problem for patients with Parkinson’s disease (PD), a disorder that, in its early stage, is dominated by basal ganglia dysfunction. Here, we test whether this action selection deficit in Parkinson’s patients arises from an impaired ability to recycle motor parameters shared across subsequent actions. Parkinson’s patients off dopaminergic medication (*n* = 16) and matched healthy controls (*n* = 16) performed a task that involved moving a handheld dowel over an obstacle in the context of a sequence of aiming movements. Consistent with previous research, healthy participants continued making unnecessarily large hand movements after clearing the obstacle (defined as “hand path priming effect”), even after switching movements between hands. In contrast, Parkinson’s patients showed a reduced hand path priming effect, i.e., they performed biomechanically more efficient movements than controls, but only when switching movements between hands. This effect correlated with disease severity, such that patients with more severe motor symptoms had a smaller hand path priming effect. We propose that the basal ganglia mediate recycling of movement parameters across subsequent actions.

## Significance Statement

The human motor system recycles motor parameters of previous actions when programming new actions, promoting efficient motor behavior. Here, we investigated the contribution of the basal ganglia to this transfer of motor parameters over subsequent actions. We assessed motor recycling by analyzing kinematic movement parameters during sequential hand movements that involved either a switch or no switch between hands. Compared with matched controls, Parkinson’s patients were impaired in transferring previously used motor parameters to new actions, but only when switching actions between hands. This suggest that the basal ganglia are important for motor recycling, and that the impaired ability of Parkinson’s patients to perform this computation may result in motor slowing.

## Introduction

The basal ganglia have an important role in organizing transitions between subsequent actions (Garr, 2019). For example, it has been suggested that the basal ganglia bind sequential motor elements into “chunks” during motor learning (Graybiel, 1998; Wymbs et al., 2012), allowing groups of individual movements to be prepared and executed as a single motor program (Graybiel, 1998; Halford et al., 1998; Wymbs et al., 2012). The basal ganglia are also involved in switching between motor and cognitive demands of a task (Aarts et al., 2010), and, more generally, in switching toward novel behavior (Redgrave and Gurney, 2006). Patients with Parkinson’s disease (PD), who have basal ganglia dysfunction (Kish et al., 1988), are behaviorally impaired in action sequencing (Benecke et al., 1987), chunking (Tremblay et al., 2010), and shifting between subsequent actions (Cools et al., 1984; Hayes et al., 1998; Helmich et al., 2009). Recent evidence has highlighted a general principle that might underlie those impairments: previous movements can influence the parameters of subsequent movements over a timescale of seconds. Multiple experiments have shown that re-using motor parameters over consecutive actions may minimize computational demands of motor planning, promoting efficient motor behavior (Jax and Rosenbaum, 2007; van der Wel et al., 2007; Hesse et al., 2008; Dixon and Glover, 2009; Tang et al., 2015). However, it is not known how the motor system re-uses motor parameters of previous actions. Here, we assess the contribution of the basal ganglia in transferring motor parameters over subsequent actions.

The neural architecture of the basal ganglia is well suited to incorporate recent motor history into newly programmed actions. First, the basal ganglia contain multiple recurrent loops between individual nuclei (Taverna et al., 2008; Redgrave et al., 2010; Díaz-Hernández et al., 2018) and between the basal ganglia and the cortex (Alexander et al., 1986). These loops are important for the formation of short-term motor memory (Berns and Sejnowski, 1998), which is necessary for relaying information from previous actions to subsequent actions and thus crucial for the efficient re-use of motor parameters across consecutive actions. Second, the direct and indirect pathways through the basal ganglia allow transitions between subsequent actions by facilitating (new) cortical motor representations through the direct pathway, while inhibiting (previous) motor representations through the indirect pathway (Downes et al., 1993; Hayes et al., 1998). This anatomic configuration appears well suited for organizing switches between subsequent actions while re-using elements of previous actions kept in motor memory. Such a process would be critical for recycling motor parameters across different actions. Here, we test the possible role of the basal ganglia in re-using motor parameters shared across subsequent actions. We consider early-stage PD as a model of predominantly basal ganglia dysfunction, testing the prediction that PD patients should be impaired in transferring motor parameters over subsequent actions, especially during action switching.

The study uses a previously validated behavioral task (van der Wel et al., 2007), showing that when participants move their hand over an obstacle, in the context of a sequence of aiming movements, they continue to make unnecessarily large movements even after the obstacle has been cleared (“hand path priming effect”). This effect is present even when participants clear the obstacle with one hand and continue making aiming movements with the other hand. This observation suggests that the hand path priming effect is driven by central motor representations, rather than by biomechanical factors. More generally, this effect suggests that humans minimize changes in motor planning between subsequent movements, sometimes at the expense of biomechanical costs. Importantly, recycling of motor parameters in this task requires previous parameters to be maintained in short-term memory and, in the case of switching actions between hands, to be generalized across different actions. Here, we compare the hand path priming effect between 16 PD patients off dopaminergic medication and 16 matched healthy controls. We expected a reduced hand path priming effect in PD patients compared with healthy controls, particularly when switching actions across hands. The prediction that hand path priming in PD patients should be particularly affected when switching actions between hands derives from the evidence that the basal ganglia play a critical role in action selection and switching (Redgrave et al., 1999; Humphries et al., 2006), in line with frequently observed behavioral impairments of PD patients when shifting between subsequent actions (Cools et al., 1984; Hayes et al., 1998; Helmich et al., 2009).

## Materials and Methods

### Participants

Sixteen patients with PD and 16 healthy controls participated in the study (Table 1). Age and gender did not differ between groups (*p* > 0.05). All participants were right-handed. We recruited controls from the local community and patients through the neurologic outpatient clinic of the Radboud University Medical Center.

**Table 1:** Participant characteristics

	PD patients	Controls
Gender (male/female)	7/9	10/6
Age (in years, mean ± SD)	60 ± 6.6	57 ± 6.5
UPDRS III (mean ± SD)	28.3 ± 7.4	**-**
Hoehn and Yahr	1 (*n* = 1) 1.5 (*n* = 1) 2 (*n* = 8) 2.5 (*n* = 5) 3 (*n* = 1)	**-**
Frontal assessment battery (FAB, mean ± SD)	17.3 ± 1.1	**-**
Disease duration (in years, mean ± SD)	3.2 ± 1.5	**-**

UPDRS III refers to the motor section of the unified PD rating scale, which has a maximum score of 108 points. The Hoehn and Yahr scale considers five disease stages; stage 2 refers to “bilateral involvement without impairment of balance.” the frontal assessment battery has a maximum score of 20 points.

Patients were included when they had PD, diagnosed according to the United Kingdom Brain Bank criteria, and asymmetric symptoms mainly on the right side of the body. In this way, we were able to test whether task performance would be different for the most-affected and least-affected side. Exclusion criteria were: severe action tremor or dyskinesias (to avoid interference with the task), cognitive dysfunction (i.e., mini-mental state examination <24), and neurologic comorbidity. Patients were at a relatively early stage of the disease. All patients were tested in a practically defined OFF state, i.e., at least 12 h after their last dose (Langston et al., 1992). The Central Committee on Research involving Human Subjects approved the experimental procedure. All participants gave written informed consent before the start of the study. The current study was not preregistered.

### Apparatus and procedure

**Figure 1.**
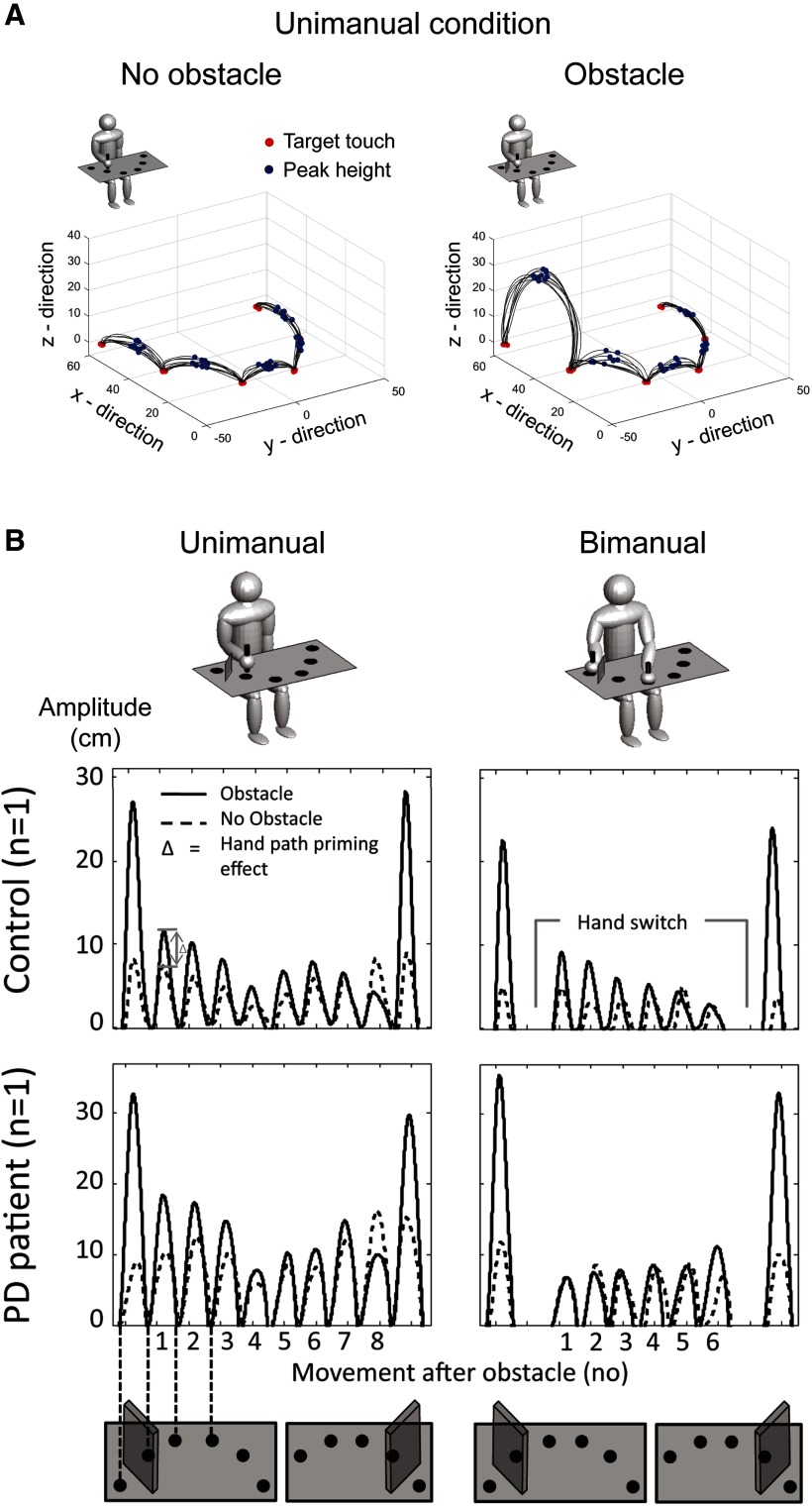
Overview of the experimental setup and example trajectories. ***A***, Example trajectories of one control participant in three-dimensional space for obstacle-absent (left panel) and obstacle-present blocks (right panel) in the unimanual condition. Participants performed jumping movements between targets (red dots). The points of peak height between the targets are marked with blue dots. Participants performed five back and forth movements in each block, as indicated by 10 connecting lines between each target. Movements were recorded with a sensor positioned between thumb and index finger of each hand, respectively. ***B***, Example trajectories for each condition, for one control participant and one PD patient, respectively. The left column shows the unimanual condition, and the right column the bimanual condition. The top row shows the experimental setup. The middle row shows the movement trajectory (averaged across all 10 repetitions) for one control participant and the lower row shows the movement trajectory for one PD patient. The movement trajectories are shown for the “no obstacle” condition (dashed lines), and for the “obstacle” condition (solid lines). The difference between these two conditions is the hand path priming effect. The empty spaces in the bimanual condition (plots on the right side) mark the hand switch. Displayed trajectories correspond to one back and forth sequence along all the targets. This plot shows that in the bimanual condition, the example control participant has a hand path priming effect for the first movement after obstacle clearance (i.e., a difference between the two lines depicting the obstacle present and obstacle absent conditions), while the example PD patient does not have a hand path priming effect.

Figure 1 shows the experimental setup, which replicated the experiment by van der Wel et al. (2007). Participants sat at a table with a board on which six targets were evenly spaced in a semicircle. Including six targets assured that participants completed multiple movements on each side of the midline, and one movement across the midline, while making sizeable arm movements within reachable space. Participants held a wooden dowel in either or both of their hands, depending on the experimental condition. The experimenter instructed participants that they were to transport this dowel from target to target using a “jumping” movement. In the experimental conditions, an obstacle stood between either the leftmost or rightmost target pair. Participants were to clear the obstacle by moving over it with the dowel. They were instructed not to move around the obstacle. No obstacle was present in the control conditions. We externally paced the movement rhythm using an auditory metronome set to 1 Hz to avoid differences in movement frequencies across participants and groups, which may influence the magnitude of the hand path priming effect (Jax and Rosenbaum, 2009). We recorded participants’ movements in three dimensions (*x*-, *y*-, and *z*-axes) with a Polhemus Liberty at a sampling rate of 200 Hz. A sensor was positioned between thumb and index finger of each hand, respectively. Before the experiments started, participants completed a practice block, lasting a few minutes, in which they moved in time with the metronome while no obstacle was present. The experiment lasted ∼32 min.

We manipulated obstacle presence (absent or present) and whether participants performed the movements with one or both hands (unimanual or bimanual). In each block, participants were asked to start the movement either on the leftmost or rightmost target. If they started on the left, in the unimanual conditions participants moved with their left hand from target to target in the rightward direction, all the way to the farthest target on the right, and then back to the left, tapping all the targets in between, to then return rightward again. When starting on the right, participants used their right hand and the sequence was reversed. For the bimanual conditions, participants held a dowel in each hand. They performed the first movement with the hand corresponding to side of the starting location. In the obstacle-present condition, this entailed moving that hand over the obstacle. They then continued the sequence with their other hand, which rested on the target next to the target pair between which the obstacle stood. Once they returned to that target, they moved over the obstacle with their initial hand again. In obstacle-present blocks, obstacles were always placed next to the starting location. Participants were asked to move back and forth five times to collect meaningful averages for each movement and to reduce the influence of start-up effects. Figure 1 shows example trajectories in three-dimensional space for obstacle-absent and obstacle-present blocks in the unimanual condition (Fig. 1*A*) and example trajectories for a control and a patient in each of the four experimental conditions (Fig. 1*B*).

### Experimental design

The experiment implemented a 2 × 2 × 2 full factorial design with factors transfer (unimanual vs bimanual), obstacle (absent vs present), and laterality (block started with left vs right hand). Each of the eight conditions was tested in two blocks, resulting in a total of 16 blocks of ∼2 min each. The order of conditions was pseudo-randomized across the experiment: the obstacle-present versus obstacle-absent conditions always alternated, and half of the participants started with the obstacle-absent condition. Each block contained at least five back-and-forth sequences consisting of 10 movements each. The hand path priming effect was quantified as the average difference in peak movement height for the obstacle-present minus the obstacle-absent conditions. This effect was calculated over 10 repetitions (two blocks × five movement sequences), separately for each of the conditions. We only used the first three movements after clearing the obstacle in our analyses. This was done for two reasons. First, by doing so, we used only movements participants made after they cleared the obstacle but before they changed their movement direction (due to reaching the last target in a given direction). Second, the results by van der Wel et al. (2007) indicated that differences in peak heights largely leveled off after the first three postobstacle movements. Including additional movements in the analysis could thus reduce the sensitivity to the hand path priming effect.

### Statistical analysis

#### Hand path priming

We statistically compared the hand path priming effect in a 2 × 2 × 2 × 3 ANOVA with within-subject factors transfer (unimanual vs bimanual), laterality (block started with left hand vs right hand), and movement (first, second or third movement after obstacle clearance), and between-subjects factor group (PD patients vs controls). We applied a Greenhouse–Geisser correction to correct the degrees of freedom whenever a violation of sphericity was indicated.

Since the magnitude of the hand path priming effect has been shown to depend on the movement amplitude of the initial obstacle-clearing movement (van der Wel et al., 2007), and PD patients and controls may exhibit different amplitudes for this initial movement, we repeated the above analysis, but normalized each participant’s hand path priming effect by the individual difference in the initial movement’s amplitude between obstacle and no-obstacle conditions.

#### Hand path priming and disease severity

We hypothesized that the magnitude of the hand path priming effect in PD patients might be influenced by the patients’ disease severity. In order to assess this relationship, we correlated disease severity, quantified as the UPDRS III score, with the hand path priming effect, separately for the first three movement after obstacle clearance in the unimanual and bimanual conditions. Since UPDRS scores are not interval scales, we computed Spearman’s rank-order correlations. Furthermore, to control for a potential influence of the movement amplitude of the initial obstacle-clearing movement, we also performed the correlation analysis on the normalized hand path priming effect.

#### Hand rotations

Next to the hand path priming effect in movement height, we also studied the effect of obstacle clearance on subsequent hand rotations (pitch and roll). To this end, we calculated the angular differences in hand orientation along the sensor’s pitch and roll axis at the movement peaks of the first three movements after obstacle clearance. Analogous to the analysis of the hand path priming effect, we subjected these data to 2 × 2 × 2 × 3 ANOVAs with within-subject factors transfer (unimanual vs bimanual), laterality (block started with left vs right hand) and movement (first, second or third postobstacle movement), and between-subjects factor group (PD patients vs controls). The purpose of this analysis was to rule out that any effects in hand path priming could be caused by systematic differences in hand rotations, which themselves may affect the height of the sensor attached to the hand.

#### Movement and dwell times

**Figure 2.**
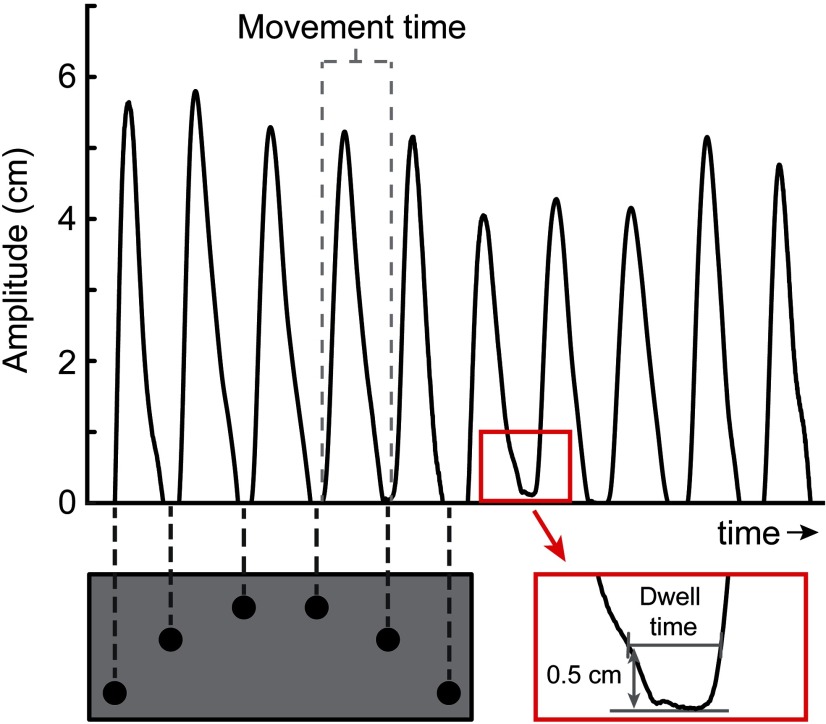
Measurement of movement and dwell times. Movement times were defined as the elapsed time between the moments when participants lifted the dowel from successive targets (gray dotted lines). Dwell times were defined as the time interval during which the dowel was within a 0.5-cm distance above the target (red box).

To further characterize participants’ behavior on the task, we additionally analyzed movement and dwell times (Fig. 2). Movement times were defined as the elapsed time between the moments when participants lifted the dowel from successive targets. For each participant, we averaged movement times across all movements within a complete back and forth movement sequence (10 movements in unimanual condition, eight movements in bimanual condition) and across all sequence repetitions in a given condition (five repetitions per block, with two blocks each), resulting in an average movement time across 100 movements in the unimanual and 80 movements in the bimanual condition, respectively. We statistically compared movement times across conditions in a 2 × 2 × 2 × 2 repeated-measures ANOVA, with within-subject factors obstacle (obstacle-present vs absent), transfer (unimanual vs bimanual), laterality (block started with left hand vs right hand), and between-subject factor group (PD patients vs controls).

Moreover, we sought to relate dwell times during which participants planned their subsequent movement to the hand path priming effect in this movement. Dwell times, i.e., the time that participants rested on each target, were defined as the time interval during which the dowel was within a 0.5-cm distance above the target, where distance is computed along the *z*-axis (i.e., height). We computed dwell times for each of the first three postobstacle targets. We then statistically analyzed the dwell time estimates in a similar ANOVA as described above. Furthermore, to relate movement preparation (dwell time) to movement plan reuse (hand path priming), we calculated correlations between the hand path priming effect and dwell times variables for the first target landing and movement after clearing the obstacle.

## Results

### Hand path priming

**Figure 3.**
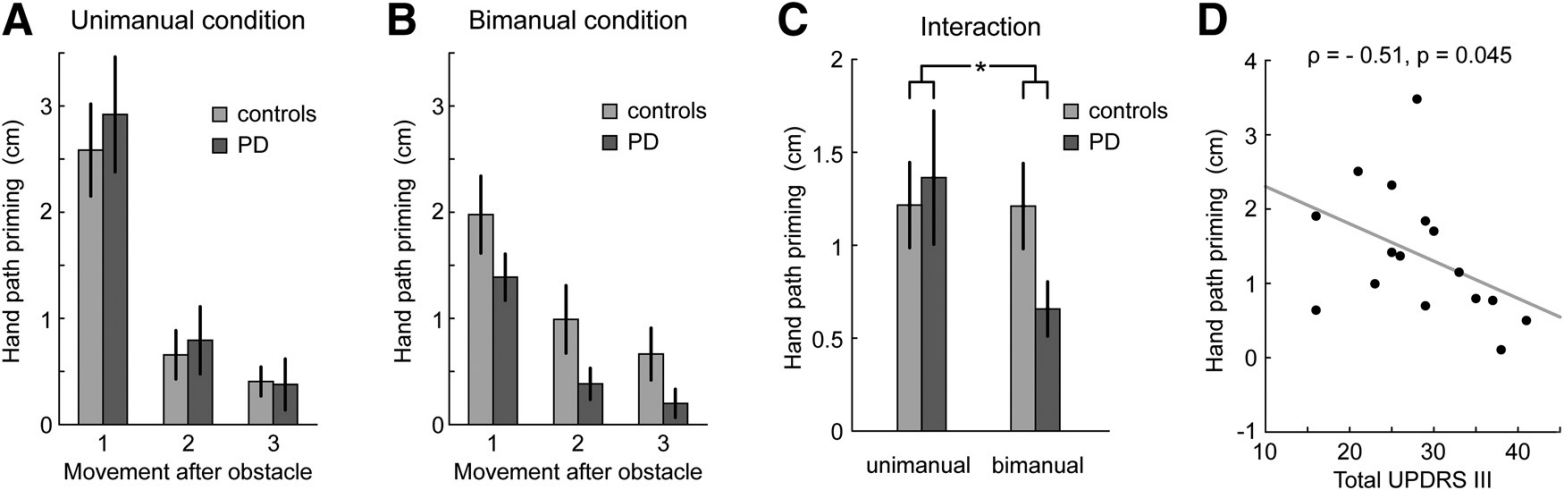
Hand path priming effect. The average difference in peak movement height between the obstacle-present and obstacle-absent conditions is plotted as a function of the movement number after clearing the obstacle, separately for the unimanual (***A***) and bimanual condition (***B***). ***C***, Significant group × transfer interaction, indicating that PD patients have a reduced hand path priming effect in the bimanual condition, but not in the unimanual condition. ***D***, Relationship between disease severity and hand path priming effect. The hand path priming effect for the first postobstacle movement in the bimanual condition decreases with increasing disease severity. Error bars depict SEMs.

Participants (i.e., both patients and controls) showed a pronounced hand path priming effect after clearing the obstacle (*F*_(1,30)_ = 43.749, *p* < 0.001; Fig. 3). This effect gradually disappeared as participants moved further away from the obstacle (main effect of movement, *F*_(1.175,35.240)_ = 79.834, *p* < 0.001). The hand path priming effect was smaller when participants switched the moving hand after clearing the obstacle (main effect of transfer, *F*_(1,30)_ = 4.683, *p* = 0.039), but it decayed more slowly (interaction between transfer and movement, *F*_(1.379,41.359)_ = 23.958, *p* < 0.001). Crucially, the hand path priming effect was smaller in PD patients than in controls, but only when patients switched between hands after clearing the obstacle (interaction between group and transfer, *F*_(1,30)_ = 4.551, *p* = 0.041). In particular, only for bimanual trials, but not for unimanual trials, there was a trend toward smaller priming in PD patients, compared with controls, which, however, did not survive the statistical threshold (bimanual: *F*_(1,30)_ = 3.122, *p* = 0.087; unimanual: *F*_(1,30)_ = 0.121, *p* = 0.731). In fact, for unimanual trials, a *post hoc* Bayesian *t* test revealed moderate evidence for the null hypothesis of no difference between PD patients and versus the hypothesis of a larger hand path priming effect in healthy participants (BF_0+_ = 3.72). There were no significant differences between movements with the left or the right hand. Together, these analyses demonstrate a reduced hand path priming effect in PD patients relative to controls, specific to the bimanual condition. Conversely, PD patients and controls exhibit a similar hand path priming effect in the unimanual condition.

Importantly, for the initial obstacle clearing movement, control participants showed a larger movement height difference (Δ clearance) between obstacle-present and obstacle-absent trials compared with PD patients (main effect of group, *F*_(1,30)_ = 8.30, *p* = 0.007). Furthermore, while control participants exhibited larger Δ clearance for movements with their dominant right compared with left hand (*F*_(1,15)_ = 9.36, *p* = 0.008), PD patients had a slight, but not significant, tendency toward smaller Δ clearance for right compared with left hand movements (*F*_(1,15)_ = 2.42, *p* = 0.14), leading to a significant group × laterality interaction (*F*_(1,30)_ = 11.76, *p* = 0.002). This likely reflects bradykinesia in the mainly right-side affected PD patients. Crucially, the difference in Δ clearance between groups was similar for the unimanual and bimanual conditions (no interaction between group and transfer, *F*_(1,30)_ = 0.87, *p* = 0.359) and thus is unlikely to explain the group × transfer interaction in the hand path priming effect. Nevertheless, we conducted a control analysis to rule out that differences in hand path priming were driven by systematic differences in the initial obstacle clearing movement. Specifically, we expressed the hand path priming effect relative to the height difference in the initial movements of obstacle-present and absent trials. Crucially, in agreement with the above results, the analysis of this relative hand path priming effect again showed a significant group × transfer interaction (*F*_(1,30)_ = 6.97, *p* = 0.013).

### Hand path priming and disease severity

We correlated the hand path priming effect for the first movement after the obstacle with disease severity (UPDRS motor score). There was a significant negative correlation for the bimanual condition (Spearman’s ρ = −0.50, *p* = 0.045; Fig. 3*D*), but not the unimanual condition. This correlation was similarly present, after controlling for individual movement height differences of the initial obstacle-clearing movement (Δ clearance) between obstacle-present and obstacle-absent trials (Spearman’s ρ = −0.50, *p* = 0.049). Thus, the greater a patient’s disease severity, the smaller his or her hand path priming effect was. There were no significant correlations between disease severity and the other movements after clearing the obstacle (*p*s > 0.3).

### Hand rotations

For the analysis of hand rotations, we excluded two participants of the control group, who showed exceptionally large differences in hand rotations between the obstacle-present and absent conditions (>60° roll or pitch difference in at least one condition). Importantly, after excluding these two participants, the group × transfer interaction in the hand path priming effect remained significant (*F*_(1,28)_ = 6.72, *p* = 0.015).

**Figure 4.**
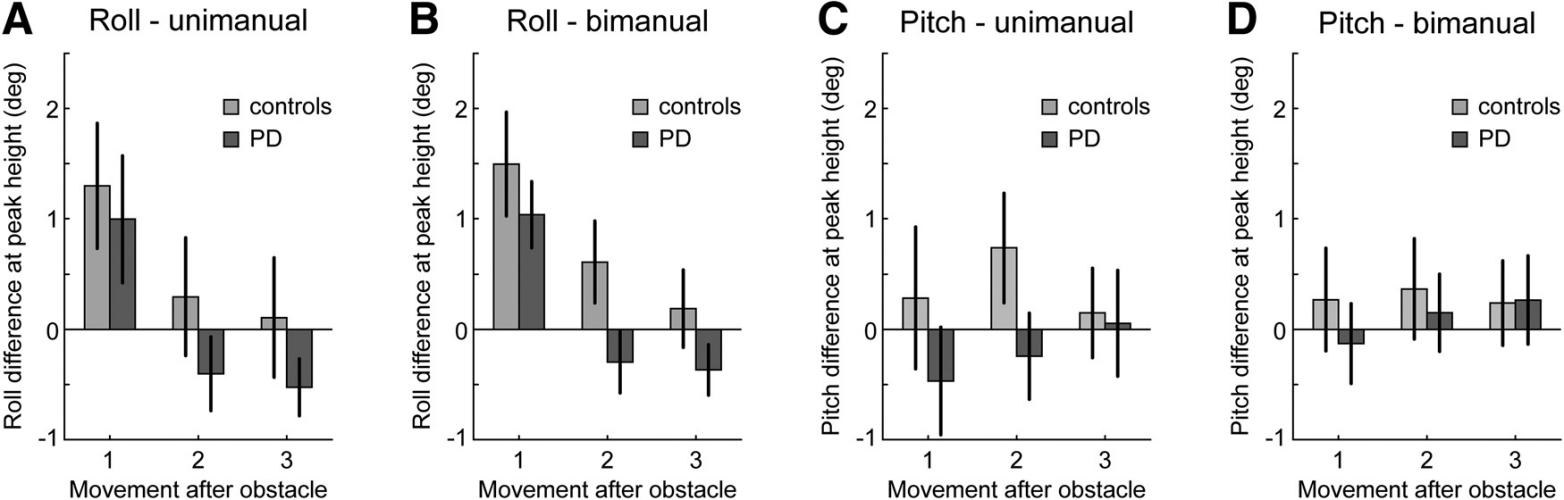
Hand rotations. Hand rotation differences between obstacle-present and absent trials at the highest points of the first three movements after clearing the obstacle. There are systematic roll rotation differences between obstacle-present and absent trials, both in the unimanual (***A***) and bimanual conditions (***B***). These differences decrease as participants move further away from the obstacle. No such patterns are found for pitch rotation differences (***C***, ***D***). There are no significant differences in hand rotations between PD patients and controls, and overall rotation differences are too small to exert a noticeable effect on the height estimate of the sensor. Error bars depict SEMs.

Overall, rotation differences between the obstacle-present and absent trials were minute (<1.5° on average; Fig. 4), and as such they were unable to account for any appreciable differences in sensor heights across conditions. Furthermore, there were no significant differences in hand rotations across groups. Therefore, it is unlikely that differences in hand path priming reported above were due to systematic differences in hand rotations, rather than a priming effect in movement amplitudes. However, although hand rotation differences were small, we found a main effect of movement on roll rotation differences (*F*_(1.18,33.12)_ = 34.31, *p* < 0.001), with roll rotation differences gradually decreasing as participants moved further away from the obstacle. This mirrored the decreasing hand path priming effects in movement amplitudes with increasing distance from the obstacle, hinting that the recycling of movement parameters might not be limited to movement amplitudes, but may include hand rotations. Consequently, in a follow-up analysis, we correlated the hand rotation differences between obstacle-present and absent trials with the hand path priming effect in movement amplitudes across participants. We found strong correlations for roll rotations, both in the unimanual and bimanual condition (unimanual: *r* = 0.66, *p* = 7.14e-5; bimanual: *r* = 0.82, *p* = 1.72e-8). Correlations were not different between PD patients and controls. There were no significant effects involving pitch rotations. Overall, this suggests that while hand rotation differences were too small to bias movement amplitude estimates in any appreciable way, in the current experiment the recycling of movement parameters was likely not limited to movement amplitudes, but extended to roll rotations of the hand. However, in contrast to priming of movement amplitudes, the effects in roll rotation were not noticeably different between PD patients and controls.

### Movement and dwell times

Participants closely followed the metronome period of 1 Hz (M_Controls_ = 976 ms; M_Patients_ = 985 ms). They exhibited shorter movement times in the bimanual compared with the unimanual condition (main effect of transfer: *F*_(1,30)_ = 32.668, *p* < 0.001) and this was especially pronounced for controls (transfer × group interaction (*F*_(1,30)_ = 6.12, *p* = 0.019). Within the bimanual condition, there was a trend toward longer movement times for PD patients compared with controls, which did not survive the statistical threshold (main effect of group, *F*_(1,30)_ = 3.75, *p* = 0.062). Importantly, there was no significant correlation between the patients’ movement times and their hand path priming effect for the first postobstacle movement (*r* = –0.39, *p* = 0.14), suggesting that the patients’ increased movement times and decreased hand path priming effects in the bimanual condition were unrelated. Furthermore, when computing movement times only for the first three postobstacle movements, for which the hand path priming effect was computed, both groups showed very similar movement times in both unimanual (M_Controls_ = 968 ms; M_Patients_ = 967 ms; *F*_(1,30)_ = 0.042, *p* = 0.84) and bimanual conditions (M_Controls_ = 1017 ms; M_Patients_ = 1005 ms; *F*_(1,30)_ = 1.466, *p* = 0.23). For detailed description of movement times, see Tables 2, 3.

**Table 2:** Movement times (in milliseconds), computed as the elapsed time between the moments when participants lifted the dowel from successive targets

	Unimanual		Bimanual	
	Obstacle**-**absent	Obstacle**-**present	Obstacle**-**absent	Obstacle**-**present
Controls	996 (2)	987 (2)	960 (2)	960 (4)
Patients	993 (3)	990 (6)	973 (4)	985 (17)

For each participant, we averaged movement times across all movements in a complete back and forth movement sequence (10 movements in unimanual condition, eight movements in bimanual condition) and across all sequence repetitions in a given condition (five repetitions per block, with two blocks each). Movement times in this table present averages over the factor laterality (left/right hand). Numbers in parenthesis indicate SEM.

**Table 3:** Movement times (in milliseconds) averaged over the first three postobstacle movements

	Unimanual		Bimanual	
	Obstacle**-**absent	Obstacle**-**present	Obstacle**-**absent	Obstacle**-**present
Controls	983 (7)	953 (8)	1036 (8)	998 (8)
Patients	996 (3)	938 (8)	1019 (9)	990 (6)

Movement times were defined as the elapsed time between the moments when participants lifted the dowel from successive targets. Numbers in parenthesis indicate SEM.

In addition to the analysis of movement times, we also analyzed dwell times on the targets between movements. Dwell times were defined as the time interval during which the dowel was within a 0.5-cm distance above the target. Similar to the hand path priming analysis, we only analyzed dwell times of the first three movement after clearing the obstacle. We found that PD patients dwelled significantly shorter on the target location compared with controls (main effect of group: *F*_(1,30)_ = 4.47, *p* = 0.043). This is likely a result of compensating for slower movements in between targets due to bradykinesia. Furthermore, we found a significant obstacle × movement interaction (*F*_(1.3,39.00)_ = 70.01, *p* < 0.001), which indicated that, specifically in the conditions in which an obstacle was present, dwell times increased as participants moved further away from the obstacle. We also observed main effects of obstacle (*F*_(1,30)_ = 95.09, *p* < 0.001) and of movement (*F*_(1.22,36.56)_ = 27,93, *p* < 0.001). Finally, there was a significant transfer × movement interaction (*F*_(1.21,36.33)_ = 6.29, *p* = 0.012), indicating that the increase in dwell times with distance from the obstacle was particularly pronounced in the unimanual condition. Importantly, there was no significant interaction involving group, suggesting that apart from generally shorter dwell times, PD patients’ dwell times followed the same patterns as those of controls. For detailed description of dwell times, see Table 4.

**Table 4:** Dwell times (in milliseconds) computed for the first three postobstacle movements

	Unimanual						Bimanual					
	Obstacle**-**absent			Obstacle**-**present			Obstacle**-**absent			Obstacle**-**present		
Movement	1	2	3	1	2	3	1	2	3	1	2	3
Controls	364 (31)	378 (30)	390 (33)	235 (27)	327 (30)	365 (34)	339 (24)	397 (29)	350 (28)	218 (26)	318 (25)	313 (28)
Patients	277 (28)	289 (29)	288 (28)	167 (16)	237 (23)	266 (28)	298 (31)	306 (25)	280 (24)	193 (28)	274 (26)	259 (23)

Dwell times were defined as the time interval during which the dowel was within a 0.5-cm distance above the target. Numbers in parenthesis indicate SEM.

To relate movement preparation to movement plan reuse, we calculated partial correlations between the hand path priming effect and dwell time variables for the first target landing and movement after clearing the obstacle, while controlling for differences in movement heights of the initial obstacle clearing movement between obstacle-present and absent trials (Δ clearance), which may both affect dwell times and the hand path priming effect. We found that in the unimanual condition the hand path priming effect was negatively correlated to dwell time (*r* = −0.48, *p* = 0.007), indicating that shorter movement preparation was associated with a stronger priming effect. In the bimanual condition this correlation was not significant over all participants (*r* = −0.19, *p* = 0.30), but was only significant for the PD patient group (*r* = −0.58, *p* = 0.024). See Table 5 for a summary of all correlations. Together, these results hint that the re-use of motor parameters from a previous action (hand path priming effect) may decrease the time to program the next action (dwell time).

**Table 5:** Partial correlations between dwell times and the hand path priming effect (first postobstacle movement), accounting for differences in movement heights of the initial obstacle clearing movement between obstacle-present and absent conditions (Δ clearance)

	Unimanual	Bimanual
All participants	ρ = –0.48 *p* = 0.007	ρ = –0.19 *p* = 0.30
Controls	ρ = –0.45 *p* = 0.09	ρ = –0.01 *p* = 0.97
Patients	ρ = –0.53 *p* = 0.04	ρ = –0.58 *p* = 0.02

## Discussion

We investigated how relatively early stage PD patients, whose pathophysiology was presumably confined to a predominant basal ganglia dysfunction, incorporate an element of a previous action (i.e., movement amplitude) into a subsequent action. To this end, we used a previously validated behavioral task (van der Wel et al., 2007), showing that when participants move their hand over an obstacle, in the context of a sequence of aiming movements, they continue to make unnecessarily large movements even after the obstacle has been cleared (hand path priming effect). Compared with healthy controls, PD patients had a reduced hand path priming effect, but only when they switched between hands. Furthermore, the magnitude of the bimanual hand path priming effect decreased with greater disease severity. This finding suggests that PD patients are impaired in adjusting previously used motor parameters to new actions, extending previous studies that regarded action switching as a transition between two discrete motor programs (Cools et al., 1984; Helmich et al., 2009). This suggests that fronto-striatal recycling of movement parameters contributes to efficient motor control. We speculate that the motor slowing characteristic of PD might result at least in part from this impaired motor recycling process.

### The hand path priming effect in PD

Both healthy controls and PD patients showed a clear hand path priming effect, indicating that they continued to make larger movements than necessary after clearing an obstacle with the same or the other hand. Participants recycled a kinematic element of the previous action, movement amplitude, when programming the next. The fact that the hand path priming effect was also present when switching between hands rules out that the priming effect is exclusively caused by mechanical factors, such as muscle relaxation (van der Wel et al., 2007). Instead, the hand path priming effect likely reflects a central property of movement planning, pertaining to movement features that generalize across different effectors and spatial locations. This inference is supported by recent data showing that different movement parameters (i.e., spatial and temporal features of movement sequences) are independently encoded in the motor system, and can be flexibly transferred from trained to novel sequences (Kornysheva and Diedrichsen, 2014). Planning of upcoming movements is more efficient by changing just those features that distinguish upcoming movements from recent movements, rather than starting “from scratch” each time a movement is required (Jax and Rosenbaum, 2007; Rosenbaum et al., 2007). Our design was optimized to test for the transfer of movement amplitude over sequential actions, while we imposed a fixed rhythm and fixed targets. However, it is likely that the recycling of movement parameters is not limited to movement amplitude in general. For instance, we found that roll rotations of the hand systematically differed between obstacle-present and absent trials for postobstacle movements, even when participants switched hands. Furthermore, this effect in roll rotations correlated with the hand path priming effect in movement amplitudes, suggesting a similar carryover for both motor parameters. However, whether the carryover in roll rotation is a mere consequence of larger movements due to amplitude priming, or whether movement parameters such as hand rotations can be independently primed remains a question for future research.

The current study controlled for several potential confounds related to comparing PD patients with controls. First, the main outcome measure (hand path priming effect) is the difference in movement amplitude between obstacle-present and absent conditions (van der Wel et al., 2007), controlling for systematic alterations in body posture between groups. Second, the differential hand path priming effect in PD is not a trivial consequence of the smaller movements performed by the PD patients (Desmurget et al., 2003). The between-groups difference in hand path priming effect was specific to the bimanual condition, and normalizing the hand path priming effect to the initial movement, which primed subsequent movements, did not change that finding. Third, movement frequency was controlled by using a metronome to time the movements to an equal rhythm in both groups. This is important, because previous work has shown that the hand path priming effect decreases with increasing intervals between subsequent actions (Jax and Rosenbaum, 2009). However, it should be noted that although both groups were generally well able to follow the imposed rhythm, there was a trend toward longer movements in the bimanual condition for PD patients compared with controls. However, this difference was very small (19 ms) and the patients’ movement times did not correlate with their hand path priming effect. Furthermore, when computing movement times only for the first three postobstacle movements, for which the hand path priming effect was quantified, both groups showed very comparable movement times. This suggests that longer movement times cannot account for the reduced hand path priming effect of PD patients in the bimanual condition. Furthermore, the similar movement and dwell times for PD patients and controls indicate that PD patients did not strongly suffer from traditional switch costs, typically characterized by increased response times following action switches. Therefore, it seems unlikely that the reduced hand path priming effect, measured in movement amplitudes, can be explained by general difficulties of switching movements between hands. Rather, the current results point toward a selective impairment in transferring motor parameters across subsequent different motor actions, which constitutes a novel type of switch cost, extending our current knowledge of switch costs in PD.

### The role of the fronto-striatal circuit in movement transitions

The impaired ability of PD patients to recycle action parameters for subsequent actions was only apparent for action switches, i.e., when participants cleared the obstacle with one hand and continued with the other. This fits with extensive literature showing that the basal ganglia are involved in switching between movements (Cools et al., 1984; Hayes et al., 1998; Helmich et al., 2009; Garr, 2019). The reduced ability to recycle motor elements from previous actions, as shown here, may force PD patients to plan new actions from scratch, causing behavioral delays (switch costs). The observed inverse relationship between the hand path priming effect (recycling) and dwell times (switch costs), when computed over all participants in the unimanual condition, is consistent with this notion. In line with the idea of impaired motor recycling in PD patients, behavioral studies have shown that PD patients re-program reaching movements during its execution (Gentilucci and Negrotti, 1999), suggesting impaired motor working memory that causes motor programs to decay during their time course. Crucially, this effect depended on the context in which the movement occurred: PD patients were perfectly able to adapt ongoing reaching movements, but they were severely impaired when adapting their movement trajectory by switching toward a new movement (Desmurget et al., 2004).

Several mechanisms may be responsible for the impaired ability of PD patients to organize transitions between consecutive actions. A first mechanism may involve the impairment of motor working memory, which is thought to rely on the presence of recurrent loops (Berns and Sejnowski, 1998). Indeed, there are multiple recurrent loops in the basal ganglia circuit, for example the short-range loop between the external globus pallidus (GPe) and subthalamic nucleus (STN; Redgrave et al., 2010), the long-range loop between the basal ganglia and the frontal cortex (Alexander et al., 1986; Redgrave et al., 2010), and recurrent connections between the thalamus and the striatum (Díaz-Hernández et al., 2018). This allows the basal ganglia to support “competitive cueing”: holding a second movement plan in abeyance while the first movement is being executed (Bhutani et al., 2013). In experimental parkinsonism, it has been shown that recurrent connections in the basal ganglia circuit are disrupted (Taverna et al., 2008) and sequential activity in the basal ganglia is abolished (Jáidar et al., 2010). Furthermore, PD patients have impaired activity in several nodes along the fronto-striatal loop, such as the supplementary motor area (SMA; Wu et al., 2010), which has a specific role in supporting bimanual sequences (Johnson et al., 1998). Thus, the loss of recurrent connections in the fronto-striatal circuit of PD patients may impair motor working memory, resulting in a rapid decay of motor parameters from previous actions. A second mechanism may involve the increased suppression of previous movements in PD. More specifically, striatal dopamine depletion in PD reduces processing in the direct pathway, which facilitates selection of new movements, and it increases processing in the indirect pathway, which inhibits previous movements (Downes et al., 1993; Hayes et al., 1998). The increased suppression of previous actions through the indirect pathway may thus prevent recycling of previous motor elements (Baladron et al., 2019). Taken together, both impaired motor memory and increased suppression of previous actions by the indirect pathway may explain the impaired recycling of previous motor elements in PD observed here.

### Inefficient motor planning in PD

Our findings indicate that PD patients were less inclined than controls to perform movements of greater amplitude than strictly necessary, although this may minimize planning costs (Jax and Rosenbaum, 2009). This fits with a large body of evidence showing that basal ganglia dysfunction in PD leads to a default amplitude setting that is lower than what is needed (Beckley et al., 1993; Horak et al., 1996; Desmurget et al., 2003). It has also been suggested that the basal ganglia estimate the “cost-to-go” during the execution of a motor task, balancing the value and costs of motor commands (Shadmehr and Krakauer, 2008). This idea is based on a study where PD patients and controls were asked to make accurate reaching movements of specified speeds (Mazzoni et al., 2007). While PD patients had normal spatial accuracy in each condition, they required more trials than controls to accumulate the required number of movements in each speed range. This was interpreted as a “reluctance” to execute movements requiring greater effort, despite preserved spatial accuracy. Our findings suggest that basal ganglia dysfunction in PD may lead to wrong priorities (Bloem et al., 2006), i.e., reduction of biomechanical costs at the expense of inefficient motor planning.

### Limitations and future research

Our study has several limitations. First, it might be argued that PD is not an adequate model of basal ganglia dysfunction, since other cerebral systems are also impaired in these patients. By including relatively early-stage PD patients (average disease duration 3.2 years) without cognitive dysfunction, we attempted to reduce the influence of such impairments, such as cortical Lewy body pathology occurring in more advanced PD (Braak et al., 2003). Surprisingly, the hand path priming effect was similar for both hands, although all patients had more motor symptoms on their right side than on their left side. This may be caused by the fact that, despite an asymmetry, 14 out of 16 PD patients had bilateral motor symptoms. Furthermore, even the clinically unaffected side was likely also influenced by the underlying disease process, as indicated by quantitative bradykinesia tests performed in limbs of PD patients that were deemed unaffected based on clinical assessments (Haaxma et al., 2010). Since there is ∼50–90% nigro-striatal cell loss at the onset of clinical symptoms (Kordower et al., 2013), this means that almost all patients already had substantial and bilateral basal ganglia dysfunction. Future studies may use neuroimaging in healthy participants, or apply focused basal ganglia lesions in primates, to further test the role of the basal ganglia in motor recycling. Second, in our design an action switch was always a switch between two different hands. Future studies may test whether the motor recycling impairment in PD is specific to a transition between effectors, or is also present when switching between different actions within one effector. Third, while the current study provides evidence for systematic differences in the recycling of motor parameters between PD patients and controls, the effect size of these differences seems to be relatively small. Since the current conclusions are based on a relatively small sample size, we deem it important that future studies will replicate and extend the current findings in larger sample sizes. This would be particularly helpful for providing a more precise estimate of the true effect size. Nevertheless, the current study, involving carefully screened PD patients, may provide a valuable starting point for understanding the role of the basal ganglia in motor recycling and the accompanied deficits in PD patients.

### Conclusion

Parkinson’s patients were impaired in recycling motor parameters shared across subsequent actions, specifically, in the context of action switching. We suggest that the basal ganglia are important for motor recycling, and that the impaired ability of Parkinson’s patients to perform this computation may result in inefficient motor behavior.

## Synthesis

Reviewing Editor: Alexxai Kravitz, Washington University in St. Louis Decisions are customarily a result of the Reviewing Editor and the peer reviewers coming together and discussing their recommendations until a consensus is reached. When revisions are invited, a fact-based synthesis statement explaining their decision and outlining what is needed to prepare a revision will be listed below.

In discussion with the two reviewers, several minor points were raised, as well as a few major concerns:

1) We all agreed that the statistical power for this study appears low. The authors should perform a power analysis to determine whether it was adequately powered to detect the main result(s). We understand collecting more data may be difficult, but if the study was under-powered the authors should either collect more data or revise the paper to clearly acknowledge this shortcoming and their reasoning for why they believe the data is worth publishing anyway. They should also clearly state whether any hypotheses were pre-registered (or not).

2) The link to the basal ganglia was indirect and tenuous. The authors should clarify what aspect of the task is affected (switching, priming, some combination?) in patients and how this is demonstrated in the presented findings. They should also discuss what, if anything, is known about brain areas and neurophysiological processes that underlie these aspects of the task.

3) There were several minor comments and requests for methodological clarifications from both reviewers that should be addressed.

See individual reviewer comments below:

*******************

Reviewer 1:

In this manuscript, the authors examine action selection impairments in patients with Parkinson’s Disease. The data show a reduced priming effect (the repeating of unnecessary movement characteristics (e.g., amplitude) that were required earlier in a motor action sequence) in patients, suggesting that the basal ganglia likely plays an important role in this repetition across actions. Overall, the study question is interesting, the manuscript is clearly written, and the results make a welcomed contribution to motor behavior in disease states. It would make the paper clearer if the authors could include more initial information (perhaps the physiological basis) about the actual/theoretical relationships between basal ganglia deficits and behavior in this specific task, as well as address a few statistical and clarification issues. My specific comments are shown below:

Major Comments:

1. It would be helpful for the authors to provide more background (actual neurophysiological evidence or theoretical framework) on why using this specific experimental paradigm is effective to study the dysfunction of the basal ganglia. In addition, the authors should expand how the priming effects specifically demonstrate that the basal ganglia mediates recycling of the movement parameters across subsequent actions.

2. Line 116: It would be helpful for the authors to add more details about the instructions given or procedures on how the participants performed the movements in the experiment. For example, how were the participants instructed to make sequential movements using the dowel? How were they instructed to pass the obstacle (go over the obstacle or go around the obstacle)? Was there any reward given to encourage the participants to make ‘good’ movements? Was there any time or velocity requirement for each trial? What axes were participants permitted to move in? If participants can leave the table x-y plane, how consistent are participants with their height deviation? Overall, a better illustration in Figure 1 would be helpful to understand the movement parameters of the experiment.

3. Line 121-122 and Figure 1: Clarification: Motion tracking was stated as based on a tracker placed on the hand. However, this marker is not shown in Figure 1, unintentionally implying emphasized importance of the dowel.

4. Were there any signs of fatigue from Parkinson’s Disease participants prior to the end of the experiment? Did this have any cumulative effect on behavior (difference between early versus late trials)?

5. Lines 153-154 and Lines 230-231: In lines 153-154, it is stated that in the bimanual condition, no hand priming effect is seen in the Parkinson’s Disease group. In lines 230-231, it is stated that a hand priming effect does exist for the Parkinson’s Disease group, but without explicit reference to under which experimental condition. Adding statements about the results of the study in the apparatus section may not be ideal, particularly without relevant statistical context. Furthermore, it would be helpful for the authors to add context about which experimental conditions are being referenced when talking about the existence of the hand priming effect.

6. Line 239 and line 327: The authors used the term “approached significance” for the effects. In the case of hypothesis testing, the results either are or are not statistically significant based on the pre-specified assumptions. Interpretation of a marginal result is nebulous. It is quite possible that the results failed to reject the null hypothesis due to under powering of the study. Was a power analysis done, prior to collection of the current data set? If so, where the sample sizes sufficient for expected effect size?

7. Line 261: In a Factor Effects Model, Main effects cannot be interpreted in isolation when interaction effects are present. Remedial measures include usage of a Cell Means Model, or simply making statements about the existence of an interaction effect, which is important in and of itself.

8. Line 262: There is no such thing as a “even more significant” effect. If an effect is statistically significant, then it exists.

Minor Comments

1. The delta is missing in Figure 1.

2. Line 147 and 148. The ‘solid lines’ and ‘dashed lines’ should be reversed.

3. Line 340. It would be helpful to state more clearly which trials were averaged.

4. Line 286. The authors excluded two subjects from the analysis of hand rotations. Do the authors have a reason why that would happen? How are the priming effects for those two subjects? Would they affect the results for the priming effects analysis?

5. Line 329. The authors showed the Parkinson’s Disease patients have longer movement times in the bimanual condition. But it is not clear to me how the longer movement times lead to the hypothesis of reduced ability to efficiently recycle movement parameters. Again, if the authors could provide more in terms of a theoretical framework, this idea may be clearer.

6. Line 195: “see above” Is there a figure/equation this is relating to?

7. Movement and dwell times section: An additional figure describing/detailing these two parameters would be beneficial.

8. Line 220: How was the 0.5 cm distance from the target calculated? For example, was this distance only in the z direction or was it calculated as a Euclidean distance?

9. Lines 251-253: The phrasing is confusing as it states “...slight, but not significant...”, but gives a significant p value (Group X Laterality Interaction effect).

10. Line 340: “over all 10 movements of each trial,” for clarity sake, please consider replacing with “over all repetitions of each condition"

*******************

Reviewer 2:

In this manuscript, the authors compare human motor performance data from control subjects and Parkinson’s Disease patients on a previously described task, the “hand path priming” task, which shows carryover of movement amplitude from one iteration to the next. The authors postulate this phenomenon is due to “recycling” of motor command information from one movement to the next. The brain circuits involved in this phenomenon are unknown. They hypothesize that the basal ganglia, which have been implicated in many aspects of motor sequencing, switching, shaping, and action selection, are an underlying substrate, and test this hypothesis indirectly, by evaluating patient motor performance in Parkinson’s Disease, in which basal ganglia dysfunction is a prominent part. They find that although hand path priming is present and of similar amplitude in controls and PD patients, PD patients show decreased hand path priming in one particular scenario - the bimanual condition. They interpret this as fitting into the idea that the basal ganglia are a critical component of switching of motor commands, but moreover that it indicates that PD patients may not be able to “recycle” motor commands effectively, making their overall movement less efficient.

The manuscript is easy to read and the figures are straightforward. As this manuscript is a human behavioral study, without imaging or electrophysiology, and has no traditional bench research, I am somewhat unsure of the match of the content of this study and the eNeuro readership. I will leave this question to the editors.

Major comments:

1. Though the task that the authors use, and the concept of hand path priming, is very compelling, it was not intuitively obvious to me (based on the author’s general concept that these “early” PD patients have selective basal ganglia dysfunction, and that the basal ganglia help streamline motor sequences), that PD patients would have completely normal hand path priming, and only have deficits in the bimanual condition. Is there any data that indicate what brain regions are recruited by their task, with or without obstacles? Are the deficits they find just a product of the fact that PD patients have trouble switching, and thus might recruit very different brain pathways to make a switch, thus abandoning the pathways that might be used for hand path priming? There is some imaging evidence that PD patients may recruit more cortical activity, for example, than healthy controls, in similar tasks. If this is what is happening (PD patients using compensatory mechanisms to achieve a task result), and its not particular to hand path priming, it is far less interesting, given the extensive evidence supporting a role for basal ganglia (and frontal cortex) in switching and sequencing tasks, and deficits in such tasks in PD.

2. The main effect the authors present and emphasize is that hand path priming is reduced in PD patients, though only in the bimanual condition. The p value for this main result is 0.04. Though I acknowledge performing extensive and well controlled behavioral studies in humans can be challenging, the N of 16 per group is probably underpowered to detect significant differences in some of the other comparisons they made. I would be more convinced that there is a true finding of hand-path priming specific to the bimanual condition if they repeated just the comparison in question in a new and larger group of subjects.

Minor comments:

1. In figure 1 the figure legend on the figure itself does not match the text of the figure legend, indicating different meanings of the dotted line and solid line.

## Author Response

We are pleased with the positive assessment of our manuscript and thank both reviewers for their constructive comments. We have addressed the reviewers’ comments in a point-by-point reply below.

In discussion with the two reviewers, several minor points were raised, as well as a few major concerns:

1) We all agreed that the statistical power for this study appears low. The authors should perform a power analysis to determine whether it was adequately powered to detect the main result(s). We understand collecting more data may be difficult, but if the study was under-powered the authors should either collect more data or revise the paper to clearly acknowledge this shortcoming and their reasoning for why they believe the data is worth publishing anyway. They should also clearly state whether any hypotheses were pre-registered (or not).

We now conducted a post-hoc sensitivity analysis, which suggests that the study was adequately powered to detect hand path priming effects, but potentially underpowered to detect some smaller effects, which may have been present (for more details see point 6 by Reviewer 1). We have revised the manuscript to acknowledge this limitation (line 573). Furthermore, we clarified that the study was not preregistered (line 102). Importantly, we would like to emphasize that the current study involved a difficult to access, carefully screened patient population, and due to the novelty of the research question, we were unable to base our sample size estimation on previously reported effect sizes. Due to the novelty of the study and the considerable difficulties in conducting such experiments in this patient population, we believe that the current study will constitute a valuable foundation for future studies and will be of high interest to the readership of eNeuro.

2) The link to the basal ganglia was indirect and tenuous. The authors should clarify what aspect of the task is affected (switching, priming, some combination?) in patients and how this is demonstrated in the presented findings. They should also discuss what, if anything, is known about brain areas and neurophysiological processes that underlie these aspects of the task.

We identify two processes that appear critical for the re-use of motor parameters across different actions, and link these processes to the anatomical organization of the basal ganglia. First, to incorporate previous motor parameters into new actions, previous parameters need to be stored in short-term motor memory. The multiple recurrent loops between individual nuclei of the basal ganglia (Díaz-Hernández et al., 2018; Redgrave et al., 2010; Taverna et al., 2008) and between the basal ganglia and the cortex (Alexander et al., 1986) can support such short-term motor memory (Berns & Sejnowski, 1998). We now emphasize this memory component more clearly in the manuscript (lines 57 and 77). Second, in order to generalize the recycling of motor parameters across different actions, motor memory needs to be transferred across subsequent actions when subjects switch between different movements or effectors. The direct and indirect pathways of the basal ganglia have been attributed a critical role in action selection and switching, facilitating (new) cortical motor representations through the direct pathway, while inhibiting (previous) motor representations through the indirect pathway (Downes et al., 1993; Hayes et al., 1998). We surmise that, when taken together, both impaired motor memory and increased suppression of previous actions by the indirect pathway may explain the impaired recycling of previous motor elements in PD observed in the current study. We now point out more clearly that the involvement of the basal ganglia in motor memory together with its prominent role in action switching, make it ideally suited to mediate the recycling of motor parameters across different actions within the current task (lines 63 and 82). Apart from the involvement of the basal ganglia in short-term motor memory and action switching, we are not aware of neuroimaging studies that specifically investigated the neural circuits involved in motor recycling. To our knowledge, the hand path priming task has never been used in neuroimaging studies. In this respect, the current study presents novel insights, which may foster new research on this topic. We emphasize the need for future neuroimaging and/or lesion studies to further investigate the involvement of the basal ganglia in motor recycling (line 569).

3) There were several minor comments and requests for methodological clarifications from both reviewers that should be addressed.

We addressed all comments and requests for methodological clarifications and adapted the manuscript accordingly. We provide detailed responses in our point-by-point replies below.

See individual reviewer comments below:

*******************

Reviewer 1:

In this manuscript, the authors examine action selection impairments in patients with Parkinson’s Disease. The data show a reduced priming effect (the repeating of unnecessary movement characteristics (e.g., amplitude) that were required earlier in a motor action sequence) in patients, suggesting that the basal ganglia likely plays an important role in this repetition across actions. Overall, the study question is interesting, the manuscript is clearly written, and the results make a welcomed contribution to motor behavior in disease states. It would make the paper clearer if the authors could include more initial information (perhaps the physiological basis) about the actual/theoretical relationships between basal ganglia deficits and behavior in this specific task, as well as address a few statistical and clarification issues. My specific comments are shown below:

Major Comments:

1. It would be helpful for the authors to provide more background (actual neurophysiological evidence or theoretical framework) on why using this specific experimental paradigm is effective to study the dysfunction of the basal ganglia. In addition, the authors should expand how the priming effects specifically demonstrate that the basal ganglia mediates recycling of the movement parameters across subsequent actions.

The goal of the current study was to investigate the role of the basal ganglia in the recycling of motor parameters across subsequent actions. The specific experimental paradigm was chosen, as it has been previously shown to elicit such a transfer of motor parameters, i.e. priming (van der Wel et al., 2007). We investigated the involvement of the basal ganglia in motor recycling by comparing healthy control participants to early-stage Parkinson’s disease (PD) patients, whom we considered as a model of predominantly basal ganglia dysfunction. Notably, it was not clear a priori whether the basal ganglia are in fact involved in motor recycling, and this posed an important empirical question. However, we do see that the anatomical organization of the basal ganglia is well suited to subserve motor recycling (now pointed out more clearly in the paragraph starting in line 52). First, in order to recycle motor parameters, previous parameters need to be stored in short-term motor memory. The multiple recurrent loops between individual nuclei of the basal ganglia (Díaz-Hernández et al., 2018; Redgrave et al., 2010; Taverna et al., 2008) and between the basal ganglia and the cortex (Alexander et al., 1986) can support such a short-term motor memory (Berns & Sejnowski, 1998). Second, to generalize the recycling of motor parameters across different actions, motor memory needs to be transferred across action switches. The direct and indirect pathways of the basal ganglia have been attributed a critical role in action selection and switching, facilitating (new) cortical motor representations through the direct pathway, while inhibiting (previous) motor representations through the indirect pathway (Downes et al., 1993; Hayes et al., 1998). Thus, the basal ganglia appear well suited for organizing switches between subsequent actions while re-using elements of previous actions kept in motor memory.

Importantly, our conclusion that the basal ganglia are involved in the recycling of movement parameters across subsequent actions relies on the assumption that PD is an adequate model of basal ganglia dysfunction. While we attempted to reduce the impact of other impairments, by including relatively early-stage PD patients, our results cannot establish with absolute certainty that the reduced hand path priming effect in PD patients is exclusively caused by dysfunctions of the basal ganglia. We acknowledge this limitation in the discussion (line 556) and emphasize the need for further neuroimaging or lesion studies to further test the role of the basal ganglia in motor recycling (line 569).

2. Line 116: It would be helpful for the authors to add more details about the instructions given or procedures on how the participants performed the movements in the experiment. For example, how were the participants instructed to make sequential movements using the dowel? How were they instructed to pass the obstacle (go over the obstacle or go around the obstacle)? Was there any reward given to encourage the participants to make ‘good’ movements? Was there any time or velocity requirement for each trial? What axes were participants permitted to move in? If participants can leave the table x-y plane, how consistent are participants with their height deviation? Overall, a better illustration in Figure 1 would be helpful to understand the movement parameters of the experiment.

We have now added an additional panel to Figure 1, in order to further illustrate the movement parameters of the experiment. We now show two example trajectories in three-dimensional space for obstacle-absent and obstacle-present blocks in the unimanual condition. This clarifies that participants performed movements in three dimensions, and moved the dowel over the obstacle (Figure 1A). We further state that the experimenter instructed participants that they were to transport this dowel from target to target using a “jumping” movement (line 118). When present, participants were to clear the obstacle by moving over it with the dowel. They were instructed not to move around the obstacle (line 121). We externally paced the movement rhythm using an auditory metronome set to 1 Hz (line 122) and we found that participants closely followed this rhythm (MControls = 976 ms; MPatients = 985 ms) (line 351). Participants moved the dowel in three dimensions (x, y and z-axis) (line 126), which is now further clarified with the addition of panel A to Figure 1. No reward was given.

Participants showed consistent movement heights, as indicated by a low within-participant variance of movement amplitudes for the first three post-obstacle movements (controls: σ2 = 1.80 cm {plus minus} 0.24 (mean {plus minus} SEM); PD patients: σ2 = 1.30 cm {plus minus} 0.26 (mean {plus minus} SEM)). Across participants, movement heights were slightly more variable (controls: σ2 = 3.16 cm {plus minus} 0.44 (mean {plus minus} SEM); PD patients: σ2 = 3.89 cm {plus minus} 0.49 (mean {plus minus} SEM)). This variability across participants is taken into account when subtracting peak movement height in obstacle-absent from obstacle-present trials.

3. Line 121-122 and Figure 1: Clarification: Motion tracking was stated as based on a tracker placed on the hand. However, this marker is not shown in Figure 1, unintentionally implying emphasized importance of the dowel.

We thank the reviewer for pointing out this potential misunderstanding. In the legend of Figure 1, we now emphasize that movements were recorded with a sensor positioned between thumb and index finger of each hand (line 156).

4. Were there any signs of fatigue from Parkinson’s Disease participants prior to the end of the experiment? Did this have any cumulative effect on behavior (difference between early versus late trials)?

There were no overt signs of fatigue in PD patients. To further investigate whether the participants’ behavior changed over time due to fatigue, we now conducted an additional control analysis. Specifically, we tested whether there was a change in movement amplitudes for the first three post-obstacle movements from the first to the second half of the experiment. One might surmise that fatigue would lead to movements with decreased amplitude in late compared to early trials. However, we found no evidence for a change in movement amplitudes across the experiment in patients (t(15) = 1.31, p = 0.21) or controls (t(15) = 0.10, p = 0.92; patients vs. controls: t(30) = 1.08, p = 0.29). This indicates that movements were relatively similar in early compared to late trials in both patients and controls, and provides evidence against notable effects of fatigue.

5. Lines 153-154 and Lines 230-231: In lines 153-154, it is stated that in the bimanual condition, no hand priming effect is seen in the Parkinson’s Disease group. In lines 230-231, it is stated that a hand priming effect does exist for the Parkinson’s Disease group, but without explicit reference to under which experimental condition. Adding statements about the results of the study in the apparatus section may not be ideal, particularly without relevant statistical context. Furthermore, it would be helpful for the authors to add context about which experimental conditions are being referenced when talking about the existence of the hand priming effect.

We apologize for this confusion. Figure 1 and the corresponding legend (line 153-154 in the original manuscript) refer to two example observers of the control and PD group, respectively. While example PD patient, shown in Figure 1, does not exhibit a hand path priming effect in the bimanual condition, there is indeed a significant (but small) priming effect across the whole group of PD patients. In the legend of Figure 1, we now clarify that the figure shows example trajectories for one control participant and one PD patient, respectively, and furthermore refer to example control participant and example PD patient, in order to demarcate these examples from the group results. We believe that illustrating examples of both the presence and absence of priming effects in example trajectories will aid the understanding of the hand path priming effect in general, and foreshadows the finding that PD patients exhibit a reduced priming effect, but only in the bimanual condition. Please also note that in line 230-231 of the original manuscript, we state the existence of an overall priming effect for all participants, regardless of group.

6. Line 239 and line 327: The authors used the term “approached significance” for the effects. In the case of hypothesis testing, the results either are or are not statistically significant based on the pre-specified assumptions. Interpretation of a marginal result is nebulous. It is quite possible that the results failed to reject the null hypothesis due to under powering of the study. Was a power analysis done, prior to collection of the current data set? If so, where the sample sizes sufficient for expected effect size?

We now refrain from the use of the term “approached significance”, and have changed the manuscript accordingly (lines 263 and 354). No a priori power analysis was performed, since we had no reliable indication of the effect size for the difference in hand path priming between PD patients and controls. We expected a very large effect size for the general hand path priming effect, based on a previous study (van der Wel et al., 2007). In line with this, in the current study, the estimated effect size for general hand path priming was indeed very large (unimanual: d = 1.42; bimanual: d = 1.38). A post-hoc sensitivity analysis revealed that, with the sample size of our study (n = 16 per group), the required effect size to detect a general hand path priming effect (one-sample t-test) with 80% power at α = 0.05 is d = 0.5 (medium effect size). Thus, the study appears to be adequately powered to detect a general hand path priming effect.

Since we assumed the hand path priming effect to be very large, we expected the difference in hand path priming between PD patients and controls to be medium to large as well. Importantly, there was no prior study on hand path priming, comparing PD patients to controls, which we could have used to estimate the effect size. A post-hoc sensitivity analysis revealed that the required effect size to detect an across-group difference in hand path priming with 80% power at α = 0.05 with a two-tailed independent two-sample t-test is d = 1.02 (large). We thus agree with the reviewer that the study might have been underpowered to detect some of the effects that were potentially present in the data, specifically in the bimanual condition for which the hand path priming effect is generally smaller (van der Wel et al., 2007). We acknowledge this more clearly in the revised manuscript and further emphasize that replicating the current results will be important (line 573). However, it should also be noted that the current study is of high novelty, and involved a difficult to access, carefully screened patient population, which complicates conducting such studies. Therefore, we expect that the current study will constitute a valuable foundation for future studies.

7. Line 261: In a Factor Effects Model, Main effects cannot be interpreted in isolation when interaction effects are present. Remedial measures include usage of a Cell Means Model, or simply making statements about the existence of an interaction effect, which is important in and of itself.

Following the reviewer’s suggestion, we now simply state the existence of a GROUP x TRANSFER interaction effect (line 288).

8. Line 262: There is no such thing as a “even more significant” effect. If an effect is statistically significant, then it exists.

We now changed the description of the statistical result to “... a significant GROUP x TRANSFER interaction”, omitting “even more significant” (line 288).

Minor Comments

1. The delta is missing in Figure 1.

Our apologies. We added the delta to Figure 1.

2. Line 147 and 148. The ‘solid lines’ and ‘dashed lines’ should be reversed.

We thank the reviewer for pointing out this mistake. We now swapped “solid” and “dashed” in the figure legend.

3. Line 340. It would be helpful to state more clearly which trials were averaged.

We now describe the averaging of movement times in more detail, both in the legend of Table 2 (line 368) and in the Materials & Methods section (line 227).

4. Line 286. The authors excluded two subjects from the analysis of hand rotations. Do the authors have a reason why that would happen? How are the priming effects for those two subjects? Would they affect the results for the priming effects analysis?

We shared the reviewer’s concern that the two participants with extreme hand rotations could have affected the statistical conclusions regarding the hand path priming effect. Therefore, we conducted a follow-up test, which showed that after excluding these two participants, the GROUP x TRANSFER interaction in the hand path priming effect remained significant (F(1,28) = 6.72, p = 0.015) (line 315). This indicates that the difference in priming between patients and controls was not critically driven by these two participants. Why the two control participants performed much more pronounced hand rotations than the remainder of the participants is difficult to assess retrospectively.

5. Line 329. The authors showed the Parkinson’s Disease patients have longer movement times in the bimanual condition. But it is not clear to me how the longer movement times lead to the hypothesis of reduced ability to efficiently recycle movement parameters. Again, if the authors could provide more in terms of a theoretical framework, this idea may be clearer.

We agree with the reviewer that the link between longer movement times and the recycling of movement parameters is not very clear. Therefore, we have now removed this speculation from the manuscript.

6. Line 195: “see above” Is there a figure/equation this is relating to?

“See above” was meant to refer to the description of the normalized hand path priming effect. We realized that this reference is confusing and now omit it in the revised manuscript.

7. Movement and dwell times section: An additional figure describing/detailing these two parameters would be beneficial.

We now added an additional figure that illustrates how movement and dwell times were extracted from the movement data (Figure 2).

8. Line 220: How was the 0.5 cm distance from the target calculated? For example, was this distance only in the z direction or was it calculated as a Euclidean distance?

We thank the reviewer for pointing out this ambiguity. The distance to the target was calculated in terms of the sensor’s height (z direction). We now clarified this in our description of the dwell time definition (line 238) and in Figure 2. As participants performed “jumping” movements, quickly lifting the dowel from the target location (see Figure 1 and 2), this provided an adequate thresholding procedure.

9. Lines 251-253: The phrasing is confusing as it states “...slight, but not significant...”, but gives a significant p value (Group X Laterality Interaction effect).

We apologize for this ambiguity. We now clarified that, in PD patients, the difference in Δ clearance between left and right hand was not significant (F(1,15) = 2.42, p = 0.14), but there was a significant GROUP x LATERALITY interaction (F(1,30) = 11.76, p = 0.002) (line 276).

10. Line 340: “over all 10 movements of each trial,” for clarity sake, please consider replacing with “over all repetitions of each condition”

We now describe the averaging of movement times more extensively, both in the legend of Table 2 (line 368) and in the Materials & Methods section (line 227). This also relates to point 3 of the reviewer.

*******************

Reviewer 2:

In this manuscript, the authors compare human motor performance data from control subjects and Parkinson’s Disease patients on a previously described task, the “hand path priming” task, which shows carryover of movement amplitude from one iteration to the next. The authors postulate this phenomenon is due to “recycling” of motor command information from one movement to the next. The brain circuits involved in this phenomenon are unknown. They hypothesize that the basal ganglia, which have been implicated in many aspects of motor sequencing, switching, shaping, and action selection, are an underlying substrate, and test this hypothesis indirectly, by evaluating patient motor performance in Parkinson’s Disease, in which basal ganglia dysfunction is a prominent part. They find that although hand path priming is present and of similar amplitude in controls and PD patients, PD patients show decreased hand path priming in one particular scenario - the bimanual condition. They interpret this as fitting into the idea that the basal ganglia are a critical component of switching of motor commands, but moreover that it indicates that PD patients may not be able to “recycle” motor commands effectively, making their overall movement less efficient.

The manuscript is easy to read and the figures are straightforward. As this manuscript is a human behavioral study, without imaging or electrophysiology, and has no traditional bench research, I am somewhat unsure of the match of the content of this study and the eNeuro readership. I will leave this question to the editors.

Major comments:

1. Though the task that the authors use, and the concept of hand path priming, is very compelling, it was not intuitively obvious to me (based on the author’s general concept that these “early” PD patients have selective basal ganglia dysfunction, and that the basal ganglia help streamline motor sequences), that PD patients would have completely normal hand path priming, and only have deficits in the bimanual condition. Is there any data that indicate what brain regions are recruited by their task, with or without obstacles? Are the deficits they find just a product of the fact that PD patients have trouble switching, and thus might recruit very different brain pathways to make a switch, thus abandoning the pathways that might be used for hand path priming? There is some imaging evidence that PD patients may recruit more cortical activity, for example, than healthy controls, in similar tasks. If this is what is happening (PD patients using compensatory mechanisms to achieve a task result), and its not particular to hand path priming, it is far less interesting, given the extensive evidence supporting a role for basal ganglia (and frontal cortex) in switching and sequencing tasks, and deficits in such tasks in PD.

We agree with the reviewer that it was not clear a priori that PD patients would have normal hand path priming in the unimanual condition, and would show reduced priming effects only in the bimanual condition. However, we expected that priming in the bimanual condition would be particularly affected, due to the critical role of the basal ganglia in action selection and switching (Humphries et al., 2006; Redgrave et al., 1999), in line with frequently observed behavioral impairments of PD patients when shifting between subsequent actions (Cools et al., 1984; Hayes et al., 1998; Helmich et al., 2009). We now clarified this prediction in the revised manuscript (line 82). We are not aware of neuroimaging studies, which investigated the brain regions recruited during motor recycling. This highlights the novelty of the current study. In the discussion section, we emphasize that future neuroimaging and/or lesion studies will be necessary to further elucidate the role of the basal ganglia in motor recycling (line 569).

The reviewer further asks whether the reduced hand path priming effect in the bimanual condition could be due to generally impaired switching performance in PD patients, potentially due to the engagement of compensatory mechanisms. We would like to note that both movement and dwell times were similar between PD patients and controls in the uni- and bimanual conditions, and the patients’ movement times did not correlate with their hand path priming effect. Thus, PD patients did not exhibit traditional switch costs, characterized by increased response times following action switches. Therefore, it seems unlikely that the reduced hand path priming effect, measured in movement amplitudes, can be explained by general difficulties of switching movements between hands. Rather, the current results point toward a selective impairment in transferring motor parameters across subsequent different motor actions, which constitutes a novel type of switch cost, extending our current knowledge of switch costs in Parkinson’s disease. We now discuss this point in the discussion section of the revised manuscript (line 485).

2. The main effect the authors present and emphasize is that hand path priming is reduced in PD patients, though only in the bimanual condition. The p value for this main result is 0.04. Though I acknowledge performing extensive and well controlled behavioral studies in humans can be challenging, the N of 16 per group is probably underpowered to detect significant differences in some of the other comparisons they made. I would be more convinced that there is a true finding of hand-path priming specific to the bimanual condition if they repeated just the comparison in question in a new and larger group of subjects.

After observing the current data, we agree with the reviewer that the study might have been underpowered to detect some of the effects that were potentially present in the data. We acknowledge this shortcoming in the limitation section in the discussion and emphasize that replicating the current results will be important (line 573). However, it should also be noted that the current study involved a difficult to access, carefully screened patient population, which complicates conducting such studies. Furthermore, the study is of high novelty, providing important insights for future studies. Therefore, we expect that the current study will constitute a valuable foundation for future research.

Minor comments:

1. In figure 1 the figure legend on the figure itself does not match the text of the figure legend, indicating different meanings of the dotted line and solid line.

We thank the reviewer for pointing out this mistake. We now swapped “solid” and “dashed” in the figure legend.
